# Update on Cuticular Wax Biosynthesis and Its Roles in Plant Disease Resistance

**DOI:** 10.3390/ijms21155514

**Published:** 2020-08-01

**Authors:** Xiaoyu Wang, Lingyao Kong, Pengfei Zhi, Cheng Chang

**Affiliations:** College of Life Sciences, Qingdao University, Qingdao 266071, Shandong, China; 2017020884@qdu.edu.cn (X.W.); konglingyao@126.com (L.K.); 2017020872@qdu.edu.cn (P.Z.)

**Keywords:** cuticular wax, wax biosynthesis, plant–pathogen interaction, plant disease resistance

## Abstract

The aerial surface of higher plants is covered by a hydrophobic layer of cuticular waxes to protect plant tissues against enormous environmental challenges including the infection of various pathogens. As the first contact site between plants and pathogens, the layer of cuticular waxes could function as a plant physical barrier that limits the entry of pathogens, acts as a reservoir of signals to trigger plant defense responses, and even gives cues exploited by pathogens to initiate their infection processes. Past decades have seen unprecedented proceedings in understanding the molecular mechanisms underlying the biosynthesis of plant cuticular waxes and their functions regulating plant–pathogen interactions. In this review, we summarized the recent progress in the molecular biology of cuticular wax biosynthesis and highlighted its multiple roles in plant disease resistance against bacterial, fungal, and insect pathogens.

## 1. Introduction

In the natural environment, plants encounter various pathogens such as viruses, bacteria, fungi, and even herbivorous insects, which seriously threaten plant growth and crop production. It was estimated that these pathogens have contributed to at least 20% of yield loss in important crops including wheat, rice, maize, potatoes, and soybeans [1,2]. Therefore, diseases caused by pathogenic microorganisms and herbivores are major factors affecting agriculture [3,4,5,6]. Unlike animals that could escape from predators and pathogens, plants must withstand pathogen attacks at the sites of growth [1,2]. Increasing evidence from studies in the model plant *Arabidopsis thaliana* revealed that plants have acquired a battery of sophisticated defense mechanisms to defend themselves against pathogen attacks during their co-evolution with various pathogens [7].

As the preformed defense, physical barriers such as spines, hairs, trichomes, thorns, and cuticles cover the aerial parts of plants [8,9]. In contrast, plant innate immunity acts as an example of induced defense. For instance, pattern-triggered immunity (PTI) was induced by the recognition of chemical molecules in the pathogen, microbial, and/or damage-associated molecular patterns (PAMPs, MAMPs, and/or DAMPs, respectively), and these chemical molecules are released during pathogen infection and recognized by pattern recognition receptors (PRRs) [10,11]. Generally, PTI is involved in a wide range of defenses, including the production of reactive oxygen species (ROS) and the cascade of mitogen-activated protein kinases (MAPKs) [12,13,14,15]. To interfere with pattern-triggered immunity, pathogens usually secrete effectors, which could be recognized by specific resistance (R) proteins, inducing effector-triggered immunity (ETI) [16,17,18,19,20]. Compared with PTI, ETI usually triggers the hypersensitive response (HR) and systemic acquired resistance (SAR) in host plants [21,22,23,24,25]. Traditionally, breeding for disease resistance in crops such as wheat, rice, potato, barley, and even soybean mainly relies on host resistance regulated by Resistance (R) genes [26]. However, in many cases, due to the variation of new pathogen strains, the resistance mediated by the R gene is less effective in the field [26]. These new pathogen strains can escape the recognition of the R gene and the R gene-mediated downstream defense [26]. Increasing evidence from studies in model plants and crops such as *Arabidopsis*, tomato (*Solanum lycopersicum*), and rice have revealed that phytohormones and their signaling pathways usually function at the downstream of pattern-triggered immunity and effector-triggered immunity to regulate plant defense, which were also considered to be effective plant defense mechanisms [27,28]. For instance, phytohormones salicylic acid (SA), jasmonic acid (JA), and ethylene (ET) are well known as the main regulators in plant defense responses [29]. In addition, phytohormones abscisic acid (ABA), auxin, gibberellin (GA), brassinosteroid (BR), and cytokinin (CK) also act as indirect factors involved in plant-pathogen interactions [27,28].

As the outmost layer exposed to the environment, cuticle covers plant aerial organs and protects plant tissues against enormous environmental challenges such as dehydration, excessive UV radiation, mechanical damage, and even pathogen infections, which have been summarized in prior reviews [30,31,32,33,34,35,36,37,38,39,40,41,42,43,44,45]. In addition to its protective roles, cuticle also gets involved in regulating plant development [33]. Although the composition of the cuticle varies among plant species, tissues, developmental stages, and even environmental conditions, plant cuticle is mainly composed of a cutin scaffold impregnated by and covered with cuticular waxes [46,47]. As a mixture of very-long-chain (VLC, >C20) fatty acids and their derivatives, cuticular waxes play multiple roles, from acting as a plant physical barrier, limiting the entry of pathogens, to functioning as a cue exploited by pathogens to initiate their prepenetration and infection processes in regulating the plant-pathogen interactions. Therefore, cuticular waxes have gained increasing attention in the study of plant disease resistance [9,48]. In this review, we summarized the recent advances in the molecular biology of cuticular wax biosynthesis and discussed their multiple roles in plant disease resistance against bacterial, fungal, and insect pathogens.

## 2. Molecular Mechanism of Cuticular Wax Biosynthesis

Plant cuticular waxes are organic solvent-extractable complex mixtures comprising very-long-chain fatty acids and their derivatives, such as aldehydes, alkanes, primary and secondary alcohols, esters, and ketones [49,50,51]. In some plant species, secondary metabolites such as flavonoids, pentacyclic triterpenoids, and tocopherols have also been identified as wax components [49,50,51]. In the model plant *Arabidopsis*, cuticular wax biosynthetic mechanisms have been characterized with the contribution of wax biosynthetic mutants and transcriptomic/proteomic analysis [49,50,51].

**Figure 1:**
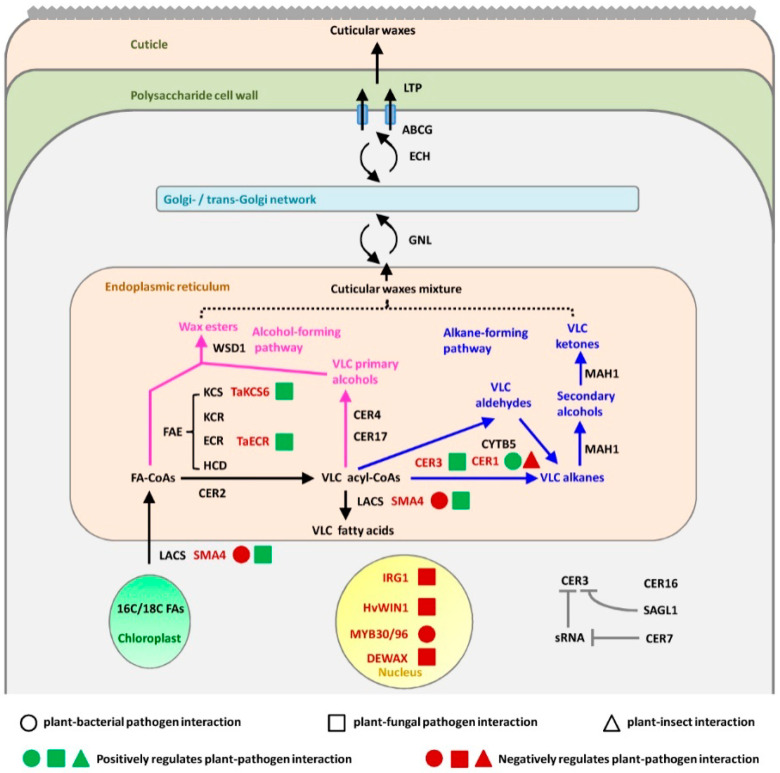
A simplified model for the cuticular wax biosynthesis and its roles in regulating plant–pathogen interactions. The biosynthesis of the cuticular wax mixture starts from the elongation of C16 or C18 fatty acid-coenzyme A (CoA) by fatty acid elongase (FAE) complex and ECERIFERUM2 (CER2) proteins. The elongated very-long-chain (VLC) acyl-CoAs are then modified into aldehydes, alkanes, secondary alcohols, and ketones by an alkane-forming pathway (shown in blue) or into primary alcohols and wax esters by an alcohol-forming pathway (shown in pink). Names shown in red denote proteins involved in the regulation of plant-pathogen interactions. For steps involving multiple paralogs, only the gene subfamily name is given in black. Circle, square, and triangle denote plant-bacterial pathogen interaction, plant-fungal pathogen interaction, and plant–insect interaction, respectively. Positive and negative regulations of plant–pathogen interaction are individually shown in green and red colors, respectively. The model for the cuticular wax biosynthesis was built on Yeast and Rose. 2013, and Lewandowska et al., 2020 [8,49].

As summarized in Figure 1, the biosynthesis of cuticular waxes in *Arabidopsis* can be divided into three steps: (1) the de novo synthesis of C16 or C18 fatty acids; (2) the extension of C16 and C18 fatty acids to form very-long-chain fatty acids (VLCFAs), which are used as direct precursors for wax synthesis in the endoplasmic reticulum (ER); and (3) the synthesis of derivatives of VLCFAs, such as aldehydes, alcohols, alkanes, ketones, and esters [49,51]. In the plastids of epidermal cells, acetyl coenzyme A (CoA) was converted into CoA-C2 by the catalysis of fatty acid synthetase complex (FAS), and after many reaction cycles, it can generate C16 or C18 acyl-acyl carrier protein (ACP), which was hydrolyzed by a fatty acyl-ACP thioesterase (FAT) to produce C16 or C18 fatty acids (Figure 1). These C16 or C18 fatty acids were activated to acyl-CoAs by the long-chain acyl-coenzyme A synthases (LACSs) and then transported to the endoplasmic reticulum (ER) [51,52]. C16 and C18 acyl-CoAs served as precursors for the formation of very-long-chain acyl-CoAs (up to C34), which was catalyzed by the enzymes of the fatty acid elongase (FAE) complex and the ECERIFERUM2 (CER2) proteins (Figure 1) [53,54]. In the FAE complex, β-ketoacyl-CoA synthase (KCS), β-ketoacyl-CoA reductase (KCR), 3-hydroxyacyl-CoA dehydratase (HCD), and enoyl-CoA reductase (ECR) catalyzed the sequential condensation/reduction/dehydration/reduction reactions in the formation of very-long-chain acyl-CoAs (Figure 1) [55,56,57,58]. These elongated very-long-chain acyl-CoAs were then modified into alkanes by the ECERIFERUM1 (CER1)/ECERIFERUM3 (CER3)/CYTOCHROME B5 (CYTB5) complex in an alkane-forming pathway, and these very-long-chain alkanes could be further oxidized to secondary alcohols and ketones by the CYP95A family cytochrome P450 enzymes MIDCHAIN ALKANE HYDROXYLASE1 (MAH1) (Figure 1) [59,60,61]. In the alcohol-forming pathway, very-long-chain acyl-CoAs were converted into the n-6 monounsaturated fatty acids by the acyl desaturase ECERIFERUM17 (CER17), which was followed by the formation of primary alcohols catalyzed by the fatty acyl-CoA reductase ECERIFERUM4 (CER4) (Figure 1) [62,63]. In addition, the *Arabidopsis* WAX SYNTHASE/ACYL-COA: DIACYLGLYCEROL ACYLTRANSFERASE 1 (WSD1) catalyzed the formation of wax esters through using acyl-CoAs and primary alcohols as precursors in the alcohol-forming pathway (Figure 1) [64]. These generated waxes components were transported from the ER to the plasma membrane (PM)via the Golgi and trans-Golgi network (TGN)-trafficking pathways, and finally exported out of the plant cell to the cuticle via the PM-localized ATP binding cassette G (ABCG) subfamily half transporters and the lipid transfer proteins (LTPs) (Figure 1) [49,50,51].

Increasing evidence from studies in *Arabidopsis* revealed that the biosynthesis of cuticular waxes is regulated by multiple transcriptional regulators [49,50,51]. As the first reported transcriptional regulator, the *Arabidopsis* APETALA2-Ethylene responsive factor (AP2-EREBP)-type transcription factor WAX INDUCER1/SHINE1 (WIN1/SHN1) and its close homologs SHN2 and SHN3 activated cuticular wax biosynthesis by upregulation of biosynthesis genes β-Ketoacyl-CoA synthase 1 (*KCS1*), *CER1*, and *CER2* [65,66]. Similarly, Myeloblastosis (MYB) family transcription factors MYB94 and MYB96 potentiated the cuticular wax biosynthesis under drought by directly activating expression of *KCS2*, *ECR*, *CER2*, and *WSD1* genes in *Arabidopsis* [67,68]. In contrast, the *Arabidopsis* AP2/ERF-type transcription factor DECREASE WAX BIOSYNTHESIS (DEWAX) was reported to negatively regulate cuticular wax biosynthesis in the light/dark cycle by directly suppressing long chain acyl-CoA synthase 2 (*LACS2*), *CER1*, and *ECR* genes [69,70]. Moreover, the biosynthesis of cuticular wax in *Arabidopsis* is regulated at the post-transcriptional and post-translational levels. For instance, ECERIFERUM7 (CER7), a core subunit of the exosome, regulated the accumulation of trans-acting small interfering RNA class of small RNAs involved in direct silencing of *CER3* in *Arabidopsis* [71]. Another recent study in *Arabidopsis* revealed that CER16, a protein with no known domains or motifs, also inhibited post-transcriptional gene silencing of CER3 to regulate alkane biosynthesis [72]. In addition, the Kelch repeat F-box protein SMALL AND GLOSSY LEAVES1 (SAGL1) mediated proteasome-dependent degradation of CER3, thereby negatively regulating cuticular wax biosynthesis in *Arabidopsis* [73].

## 3. Regulation of Plant–Bacterial Pathogen Interaction by Cuticular Waxes

On the plant cuticle, bacterial pathogens usually produce extracellular polymeric substances and form large aggregates to help them to withstand the harsh surrounding conditions. It is well known that the bacterial pathogen *Pseudomonas syringae* can produce syringafactin, a compound with surfactant properties, to facilitate its motility and increase the permeability of *Arabidopsis* cuticle [74]. Increasing evidence has revealed that the survival and infection of bacterial pathogens on plant surfaces are affected by the integrity and permeability of the plant cuticular wax layer. A more permeable plant cuticular wax layer could lead to either enhanced resistance or susceptibility to pathogen infections [48]. For instance, *Arabidopsis sma4* (symptoms to multiple-regulated avr4) is a loss-of-function mutant of the *SMA4* gene, which encodes the cuticular wax biosynthetic component LACS2 [75]. Tang et al. found that the *sma4* mutant exhibited enhanced susceptibility to the hemibiotrophic bacterial pathogen *Pseudomonas syringae* pv *tomato* strain DC3000 (*Pst* DC3000) but displayed enhanced resistance against the necrotrophic fungal pathogen *Botrytis cinerea* (*B. cinerea*) [76].

MYB family transcription factors have been reported to be involved in plant cuticular wax biosynthesis and disease resistance against bacterial pathogens [48,51]. For instance, *Arabidopsis* transcription factor MYB30 functions as a positive regulator of a cell death pathway, conditioning the hypersensitive response [77]. Raffaele et al. demonstrated that the exacerbated hypersensitive response phenotype of MYB30-overexpressing *Arabidopsis* lines was altered by the loss of function of the acyl-ACP thioesterase gene acyl-ACP thioesterase B *(FATB)*, which causes severe defects in the supply of fatty acids for the biosynthesis of very-long-chain fatty acids, suggesting that MYB30 modulates hypersensitive response via controlling the biosynthesis of very-long-chain fatty acids in *Arabidopsis* [77]. Similarly, Zhang et al. reported that ectopic expression of apple (*Medicago truncatula*) *MdMYB30*, which encodes an R2R3 MYB transcription factor, in *Arabidopsis* increased the transcription levels of wax biosynthesis-related genes, including wax synthesis regulatory gene 1 (*AtWRI1*), *AtWIN1*, *AtKCS1*, acyl-CoA binding protein 1 (*AtACBP1*), *AtLACS2*, *AtSHINE2*, and *AtSHINE3* [78]. The accumulation of wax compositions, such as C29 alkanes, C31 alcohols, C29 aldehydes, C16 fatty acids, C29 ketones, and C29 and C30 esters were significantly enhanced in *MdMYB30*-ectopic-expression *Arabidopsis* lines [78]. Interestingly, MdMYB30 also contributed to the increased resistance against *Pst* DC3000 in *Arabidopsis*, suggesting that the changed epicuticular wax content and composition might cause disease resistance against the bacterial pathogen *Pst* DC3000 [78]. In addition, MYB96, another MYB transcription factor (TF) in *Arabidopsis*, directly binds to the promoters of wax biosynthetic genes including *KCS1*, *KCS2/DAISY*, *KCS6*, *KCR1*, and *CER3*, and actives the expression of these genes under drought- and ABA-inducible conditions [79]. Notably, MYB96 activation tagging *Arabidopsis* lines showed increased wax accumulation and enhanced resistance to *Pst* DC3000 by potentiating the SA biosynthesis [80]. However, the accumulation of cuticular wax components does not necessarily contribute to the plant disease resistance against bacterial pathogens. For instance, VLC alkanes were accumulated but the susceptibility to *Pst* DC3000 was enhanced in the *Arabidopsis* lines over-expressing *CER1* [59]. Interestingly, increasing evidence from studies in fungal pathogens *Blumeria* and *Colletotrichum* revealed that certain wax components such as very-long-chain aldehyde and terpenoids could be exploited by certain fungal pathogens to trigger their infection processes, but other components, including free fatty acid RR (resistance-related) metabolites, contribute to plant resistance against fungal pathogens. Therefore, characterizing the function of specific wax components in regulating bacterial growth and infections, as well as plant defense responses, might contribute to understanding the roles of cuticular waxes in the regulation of plant–bacterial pathogen interactions in the future research.

## 4. Regulation of Plant–Fungal Pathogen Interaction by Cuticular Waxes

During the infection process, fungal pathogens could synthesize and secrete hydrolytic enzymes such as cutinases and lipases to degrade the cuticular wax layer [81]. For instance, cutinase2 (*CUT2*) gene in rice blast (*Magnaporthe grisea*) is activated during appressorial development and fungal penetration [82]. During these processes, fungal pathogens would be recognized by the plant immune systems and trigger the immune responses. Several *Arabidopsis* plants over-accumulating fungal cutinase exhibited increased cuticle permeability and enhanced resistance to fungal pathogens. For example, the *Arabidopsis* AP2/ERF-type transcription factor DECREASE WAX BIOSYNTHESIS (DEWAX) negatively regulates cuticular wax biosynthesis by suppressing cuticular wax biosynthesis genes (*CER1*, *LACS2*, ATP-citrate lyase A2 and *ECR*) [83]. Interestingly, over-expression *DEWAX* in *Arabidopsis* led to enhanced disease resistance against grey mildew (*Botrytis cinerea*) [69]. Further analyses revealed that DEWAX acts as a transcriptional activator, binding to the promoters of defense-related genes including plant defensin 1.2a (*PDF1.2a*), indole glucosinolate O-methyltransferase 1 (*IGMT1*), and peroxidase 37 (*PRX37*), and upregulating the expression of these genes in *Arabidopsis* [69]. These results suggest that cuticle wax biosynthesis genes could regulate plant disease resistance through direct targeting defense-related genes. Indeed, the *Arabidopsis sma4* mutant plants display increased resistance to grey mildew (*B. cinerea*), and these processes were independent of jasmonic acid (JA)- and ethylene (ET)-signaling pathways [76].

Interestingly, *Arabidopsis* mutants such as long-chain acyl-CoA synthetase-2 (*lacs-2*) and *-3*, and myeloblast transcription factor 96 (*myb96*) with increased cuticle permeability exhibited enhanced disease resistance against grey mildew (*B. cinerea*) [76,80]. L’Haridon et al. reported that a permeable cuticle in *Arabidopsis* is associated with the release of reactive oxygen species (ROS) and induction of innate immunity, demonstrating the importance of fungal suppression by reactive oxygen species formation [84]. In contrast, Cui et al. demonstrated that *Botrytis* immunity conferred by cuticle permeability can be genetically uncoupled from phosphatase2c-regulated abscisic acid (ABA) sensitivity but requires negative regulation of a parallel ABA-dependent cell death pathway [85]. In addition, several studies revealed that cuticular wax accumulation also contributes to disease resistance against fungal pathogens. For instance, Zhang et al. showed that the apple (*M. truncatula*) transcription factor MdMYB30 could bind to the promoter region of *MdKCS1* to activate its expression and induce wax biosynthesis [78]. Notably, the infection of apple canker pathogen *Botryosphaeria dothidea* could induce the accumulation of wax crystals and transcription of pathogenesis-related genes, such as *MdNPR1*, *MdPR1*, *MdPR5*, *MdEDS1*, and *MdPAL* at *B. dothidea* injection sites in *MdMYB30*-overexpression apple lines [78]. Consistently, *MdMYB30*-overexpression transgenic apple calli exhibited strengthened resistance against apple canker (*B. dothidea*). These results indicated that MdMYB30 positively modulates waxes biosynthesis of apple fruit and enhances apple resistance to certain fungal pathogens [78].

Powdery mildew caused by the fungal pathogen *Blumeria graminis* is a devastating disease in barley and wheat. Increasing evidence has revealed that *B. graminis* could utilize plant cuticular wax components to initiate their prepenetration processes such as conidial germination and appressorial development [86,87,88,89,90,91,92]. Hansjakob et al. reported that very-long-chain aldehydes could stimulate the in vitro conidial germination of *B. graminis* in a dose-dependent manner [86,87]. Recently, Wang et al. and Kong et al. reported that the silencing of the wheat 3-ketoacyl-CoA synthase *(TaKCS6)* and enoyl-CoA reductase (*TaECR*) led to the reduction of cuticular wax load and attenuated conidial germination of *Blumeria graminis* f.sp. *tritici* (*Bgt*). Interestingly, the *Bgt* germination penalty on the *TaKCS6-* or *TaECR*-silenced wheat plants could be fully restored by the application of wild-type cuticular waxes or very-long-chain aldehydes, suggesting that the very-long-chain aldehydes were the wax signals provided by *TaKCS6* and *TaECR* for stimulating *Bgt* conidia germination in bread wheat [91,92]. In *Arabidopsis*, Inada and Savory reported that the powdery mildew pathogen *Golovinomyces orontii* (*G. orontii*) could infect the mature rosette leaves of *Arabidopsis*, but its prepenetration processes such as conidial germination and appressorial formation were strongly inhibited on stems, fruits, and roots of *Arabidopsis* [93]. In addition, they found that inhibition of prepenetration processes of powdery mildew pathogen *G. orontii* on *Arabidopsis* stems was more severe in the mutant *cer3* but not in *cer1*, which is consistent with the fact that *CER1* gets involved in the biosynthesis of very-long-chain alkanes, but *CER3* mediates the formation of very-long-chain aldehydes [93]. Therefore, stimulating germination of powdery mildew conidia by very-long-chain aldehydes might be conserved during the interactions of powdery mildew pathogens with monocots and dicots.

Fusarium head blight (FHB) caused by the fungal pathogen *Fusarium graminearum* is another devastating disease in wheat and barley. Silencing of barley WAX INDUCER1 (*HvWIN1*), a gene essential for the regulation of cuticular wax biosynthesis, resulted in enhanced susceptibility to FHB [94]. Further study showed the contents of free fatty acid RR (resistance-related) metabolites such as linoleic and palmitic acids, and the transcript abundance of genes involved in cuticular wax biosynthesis, including *CYP86A2*, *CYP89A2*, and *LACS2*, were significantly reduced in the *HvWIN1*-silenced barley leaves upon pathogen inoculation, suggesting that HvWIN1 regulates the expression of free fatty acid biosynthesis genes to reinforce cuticle to resist head blight in barley [94].

As a typical fungal pathogen, rust has evolved special mechanisms for invading plants. Upon rust infection, fungal urediniospores need to attach to the surface of leaves and subsequently form germ tubes. In general, rust pathogen requires specific plant surface topography and chemical signals to trigger the formation of prepenetration structures [95,96]. Barrel medic (*Medicago truncatula*) gene *IRG1*, encoding a Cys(2) His(2) zinc finger transcription factor, contributes to the plant nonhost resistance to fungal pathogens. *Phakopsora pachyrhizi* and *Puccinia emaculata* are the main pathogens causing soybean (*Glycine max*) and switchgrass (*Panicum virgatum*) rust, respectively [97,98]. The barrel medic inhibitor of rust germ tube differentation1 (*irg1*) mutant showed retarded prepenetration structures of two rust pathogens, *P. pachyrhizi* and *P. emaculata*, and one anthracnose pathogen, *Colletotrichum trifoli* [99]. Further analyses revealed that abaxial epicuticular wax crystals were completely lost and surface hydrophobicity was reduced in barrel medic (*M. truncatula*) *irg1* mutant [99]. Meanwhile, the compositions of epicuticular waxes were changed in *irg1* mutant, with fewer C30 primary alcohols as well as more C29 and C30 alkanes [99]. Transcriptome analysis found that ECERIFERUM 4, an enzyme involved in primary alcohol biosynthesis, and MYB96, a major transcription factor regulating wax biosynthesis, were downregulated in *irg1* mutant, suggesting that IRG1 executes a regulating role in the biosynthesis of epidermal wax, which might affect the germination and appressorial formation of nonhost fungal pathogens in barrel medic (*M. truncatula*) [99]. Similarly, a barley gene, *CYP96B22*, encoding a putative cytochrome P450 monooxygenase, was also known to be involved in cuticular wax biosynthesis [100]. The expression of *CYP96B22* was induced by the inoculation of rice blast *Magnaporthe orzae* at the nonhost barley leaves [100]. Meanwhile, the silencing of *CYP96B22* using barley stripe mosaic virus-mediated gene silencing (BSMV-VIGS) led to a decrease in penetration resistance of barley plants to host and nonhost isolates of blast *Magnaporthe*, suggesting that CYP96B22 plays a role in the disease resistance against rice blast *M. orzae* infection [100]. Further studying the roles of IRG1 and CYP96B22 might help us to improve understanding of the significance of cuticular wax deposition on plant disease resistance against fungal infection in the future.

## 5. Regulation of Plant–Insect Interaction by Cuticular Waxes

Growing in their natural environment, plants are usually attacked by a variety of herbivorous insects. It was estimated that insect infestation leads to yield losses of more than 20% in wheat, soybean, and cotton [101]. As summarized in a prior review, plant–insect interactions are regulated by plant cuticular waxes at multiple levels [102]. First, cuticular waxes contribute to the slippery nature of plant surfaces, thus affecting plant-insect interactions [96]. For instance, cuticular waxes coating stems of many *Macaranga* ant plants (*Euphorbiaceae*) contain a large amount of triterpenoids, rendering the surface very slippery for most insects and allowing its symbiotic ants to survive in a competitor-free environment [103,104]. Second, certain cuticular wax components such as long-chain alkanes could be exploited by insects for host selection [105,106,107,108,109]. Spencer J.L. reported that the addition of long-chain alkanes in sinigrin and cabbage homogenates could stimulate oviposition by the diamondback moth *Plutella xylostella* [110]. Third, egg deposition of insects could significantly affect the composition of plant cuticular waxes, which could be sensed by egg parasitoids [111]. Blenn et al. reported that oviposition by the large cabbage white butterfly *Pieris brassicae* led to changes in the amounts of the wax composition such as the fatty acids tetracosanoic acid and tetratriacontanoic acid in *Arabidopsis*, and that the tetracosanoic acid could attract the egg parasitoid *Trichogramma brassicae* [111].

In addition to studies on the correlation of insect behaviors and plant wax compositions, the plant transcriptomic analysis also contributes to our understanding of the regulation of plant-insect interaction by cuticular waxes. For instance, tea green leafhopper, *Empoasca* (*Matsumurasca*) *onukii* Matsuda, is one of the most harmful pests to tea plants (*Camellia sinensis*), seriously threatening tea yield and quality [112]. A recent transcriptomic analysis revealed that genes involved in cuticle wax biosynthesis were significantly upregulated by tea green leafhopper infestation in tea plants [112]. In particular, the transcription level of a *CER1* homolog involved in the formation of cuticular wax alkane was most significantly elevated, and C29 alkanes in tea leaf waxes were increased [112]. These results suggested that the *CER1* homolog plays a pivotal role in tea wax alkane formation and is probably involved in responding to tea green leafhopper and other environmental stresses. Therefore, it is intriguing to characterize the function of other cuticular wax biosynthesis genes in regulating plant-insect interaction in future research.

## 6. Conclusions and Perspectives

In this review, we discussed the recent progress in the understanding of plant cuticular wax biosynthesis and its important roles in regulating plant-pathogen interactions (as summarized in Figure 1). Although past decades have seen a great advance in understanding the function of cuticular wax biosynthesis genes in model plants, we still have a long way to go towards fully understanding the biosynthesis of plant cuticular wax. For instance, exact enzymes mediating biosynthesis of certain cuticular wax components such as very-long-chain aldehydes need to be identified. Furthermore, biosynthesis of cuticular wax shares many precursors and energies with metabolisms of other substances such as saccharides, lipids, and even amino acids, but how plants orchestrate these biosynthetic processes is still unknown. In addition, increasing evidence has revealed that the biosynthesis of cuticular wax is regulated by developmental signals and environmental conditions, and their underlying mechanisms also remain to be disclosed.

As summarized in Table 1, many cuticular wax biosynthesis genes get involved in plant–pathogen interaction. As we know, the mixture of cuticular waxes is mainly composed of very-long-chain fatty acids and their derivatives, such as aldehydes, alkanes, primary and secondary alcohols, esters, and ketones, but the exact cuticular wax components responsible for limiting pathogen infection or inducing plant defense responses remains to be identified. Although it was demonstrated that very-long-chain aldehydes function as wax signals to trigger the conidial germination and appresorial development of *B. graminis* in barley and wheat, wherein the chemical regulation seems to be very specific between plant-pathogen interactions. Therefore, identifying these cuticular wax components that induce plant defense or pathogen infection and characterizing their underlying mechanisms would certainly improve our understanding of the function of cuticular wax biosynthesis in plant disease resistance. In addition, most of our knowledge about plant cuticular wax biosynthesis and their roles in plant disease resistance come from the study of the model plant-pathogen interaction, such as *Arabidopsis* and the fungal pathogen *Botrytis cinerea*, or bacterial pathogen *Pst* DC3000. However, the roles and mechanisms of cuticular waxes in regulating crop-pathogen interactions remain to be explored in future research.

Our knowledge of cuticular wax biosynthesis and their roles in plant–pathogen interactions could bring us valuable information to develop new strategies for crop protection. For instance, based on an understanding about the cuticular wax biosynthetic pathway and the function of exact cuticular wax components in plant-pathogen interaction, we could employ genome-editing systems such as the clustered regularly interspaced short palindromic repeats and CRISPR associated protein 9 (CRISPR-Cas9) system to create genome-edited crops producing the “ideal” layer of cuticular waxes without wax cues exploited by pathogens [113]. In addition, the knowledge of cuticular wax biosynthesis and their functions in plant-pathogen interaction would help us to synthesize the “elicitor” cuticular wax components to prime plant defense responses and limit pathogen infection.

## Figures and Tables

**Table 1:** Cuticular wax biosynthesis genes involved in plant-pathogen interactions.

Gene Name	Gene Product	Gene Product Family	Plant Species	Function of Gene Product	Involvement of Gene Product in Plant-Pathogen Interaction and Evidence	Reference
*DEWAX*	DEWAX	AP2/ERF-type transcription factor	*Arabidopsis thaliana*	Transcriptional suppression of cuticular waxes biosynthesis genes	DEWAX acts as transcriptional activator of defense-related genes and positively regulates disease resistance against *Botrytis cinerea.*	[69]
*SMA4*	LACS2	Long chain acyl-CoA synthetase	*Arabidopsis thaliana*	Biosynthesis of C16 or C18 acyl-CoAs	*sma4* mutant exhibited enhanced susceptibility to bacterial pathogen *Pst* DC3000 but enhanced resistance against fungal pathogen *B. cinerea.*	[76]
*MYB30*	MYB30	R2R3-type MYB family transcription factor	*Arabidopsis thaliana*	Transcriptional activation of cuticular waxes biosynthesis genes	Hypersensitive response was exacerbated in *MYB30*-overexpressing lines.	[77]
*MdMYB30*	MdMYB30	R2R3-type MYB family transcription factor	*Malus domestica*	Transcriptional activation of cuticular waxes biosynthesis genes	Ectopic expression of *MdMYB30* in *Arabidopsis* increases resistance to *Pst* DC3000.	[78]
*MYB96*	MYB96	R2R3-type MYB family transcription factor	*Arabidopsis thaliana*	Transcriptional activation of cuticular waxes biosynthesis genes	MYB96 activation-tagging *Arabidopsis* lines exhibited enhanced resistance to *Pst* DC3000 by potentiating SA biosynthesis.	[80]
*CER1*	CER1	VLC-aldehyde decarbonylase putative	*Arabidopsis thaliana*	Formation of VLC alkanes	The susceptibility to *Pst* DC3000 were enhanced in the *Arabidopsis* plants over-expressing *CER1.*	[59]
*TaKCS6*	TaKCS6	3-Ketoacyl-CoA synthase	*Triticum aestivum*	Biosynthesis of VLC acyl-CoAs	Silencing of *TaKCS6* attenuated *Bgt* conidia germination in bread wheat.	[91]
*TaECR*	TaECR	Enoyl-CoA reductase	*Triticum aestivum*	Biosynthesis of VLC acyl-CoAs	Silencing of *TaECR* attenuated *Bgt* conidia germination in bread wheat.	[92]
*CER3*	CER3	VLC-acyl-CoA reductase putative	*Arabidopsis thaliana*	Formation of VLC alkanes	The inhibition of prepenetration processes of *Golovinomyces orontii* on *Arabidopsis* stems is more severe in the mutant *cer3.*	[93]
*HvWIN1*	HvWIN1	AP2-EREBP-type transcription factor	*Hordeum vulgare*	Transcriptional activation of cuticular waxes biosynthesis genes	Silencing of *HvWIN1* resulted in enhanced susceptibility to FHB.	[94]
*IRG1*	IRG1	Cys_2_His_2_ zinc finger transcription factor	*Medicago truncatula*	Formation of epicuticular wax crystals	*irg1* mutant showed retarded prepenetration of two rust pathogens and one anthracnose pathogen.	[99]
*CYP96B2*	CYP96B2	Cytochrome P450 monooxygenase putative	*Hordeum vulgare*	Cuticular waxes biosynthesis	Silencing of *CYP96B22* led to a decrease in penetration resistance of barley plants to blast pathogen *Magnaporthe*.	[100]
